# Heavy metals and parasitological infection associated with oxidative stress and histopathological alteration in the *Clarias gariepinus*

**DOI:** 10.1007/s10646-022-02569-9

**Published:** 2022-07-15

**Authors:** Heba N. Gad EL-Hak, Mahi A. Ghobashy, Farida A. Mansour, Nahla S. El-Shenawy, Marwa I. Saad El-Din

**Affiliations:** Zoology Department, Faculty of Science, Suez Canal University, Ismailia, 41522 Egypt

**Keywords:** *Clarias gariepinus*, Metal accumulation, Oxidative stress, Seasonal variation, Al Sharkia Government

## Abstract

The goal of this study was to assess the harmful effects of heavy metal accumulation on *Clarias gariepinus* (catfish) in two different polluted areas in the Al Sharkia governorate and assess the impact on oxidative stress and histological changes. The results revealed a highly significant difference in heavy metal levels in the water and inside fish tissues (liver and gonads) between the two sites. The total prevalence of parasitic infection was at the highest percentage in area B, in addition to severe histopathological damage to the liver and the gonads. Findings show that the total prevalence of parasitic infection is associated with uptake of metals, depleted antioxidant activity, and incidence of lipid peroxidation in tissue.

## Introduction

Water pollution is a major problem in Egypt’s freshwater canals, which is one of the country’s environmental and public health challenges (Luo et al. 2020). Pollution is generally associated with industrial waste, which is one of these areas’ ecological problems (Goel 2006). Heavy metals are the most common pollutants and are toxic in coastal areas at even low concentrations (Soliman et al. 2015). Al Sharkia Governorate Canal is one of these canals that receive polluted water rich in heavy metals from many sources, including industrial and domestic effluents (Hussien et al. 2020). The toxic effects of heavy metals in fish induce alterations both at the structural and functional levels of different organs (Authman et al. 2015). Metal accumulation in fish promotes reduction-oxidation processes, that create reactive oxygen species (ROS), which can cause oxidative stress, and morphological, and biochemical changes in their tissues (Biller and Takahashi 2018). The relationship between parasitism and pollution, especially in aquatic habitats and the role of parasites as bioindicators of heavy metal pollution, is not simple and, in essence, involves a double-edged phenomenon, in which parasitization may increase host susceptibility to toxic pollutants or in which pollutants may result in an increase (or in some decrease) in the prevalence of certain parasites (Sures et al. 2017). On the other hand, biomarkers can provide extra physiologically and ecologically relevant information for the development of appropriate environmental management recommendations (Adams et al. 2001). As a result, fish biomarkers are required for monitoring environmental changes and determining the impact of contaminated water on fish (Yancheva et al. 2016).

African catfish (*Clarias gariepinus*) is a valuable commercial fish since it is one of the richest and least expensive sources of protein and omega fatty acids (Osibona et al. 2009). African catfish have a relatively high abundance and propagation in the Al Sharkia Governorate canal (Farrag et al. 2019). Generally, the consumption of African catfish from the Al Sharkia Governorate canal is considered a health problem when used for human consumption (El-Shenawy et al. 2021a). In humans, heavy metals have been related to liver and kidney damage, cardiovascular disease, and even death (Pandey and Madhuri 2014). Elawady et al. (2019) stated that the levels of heavy metals in African catfish samples from Al Sharkia were higher than those in water samples. As a consequence, it might be a useful model for researching reactions to various environmental contaminants (Farombi et al. 2008). Metals are well-known inducers of oxidative damage in fish, reflecting metal contamination of the aquatic ecosystem (Mahboob 2013). The fish’s endogenous antioxidant can neutralize the oxidant effect of free radicals, including some natural and other substances (Hamid et al. 2010). Parasites respond to heavy metal pollution either as effect indicators or stress effectors. Heavy metal concentrations in surface water often exceeded the World Health Organization’s regulatory limits. Parasites have been shown to have thousands of times higher heavy metal concentrations than their fish hosts (Sures 2003). Thus, the objective of the present study was to evaluate the responses of African catfish (*Clarias gariepinus*) liver and gonad oxidant status as a bioindicator to the various environmental heavy metal pollutants.

## Materials and methods

### Chemicals and equipment

Merck Germany provided the standard chemical for the target heavy metals with the greatest purity level (99.98 percent). For sample digestion, ultra-pure HNO_3_ was employed. All the other acids and compounds were pure and came from Merck or Scharlau in Germany or Spain, respectively. High-quality commercial kits (Biodiagnostic Co; 29 El-Tahrir St., Dokki, Giza, Egypt) were used to measure aspartate-amino transaminase (AST), alanine amino transaminase (ALT), lipid peroxidation (LPO), glutathione reduced (GSH), superoxide dismutase (SOD), and catalase (CAT).

### Description of the investigated area

**Fig. 1:**
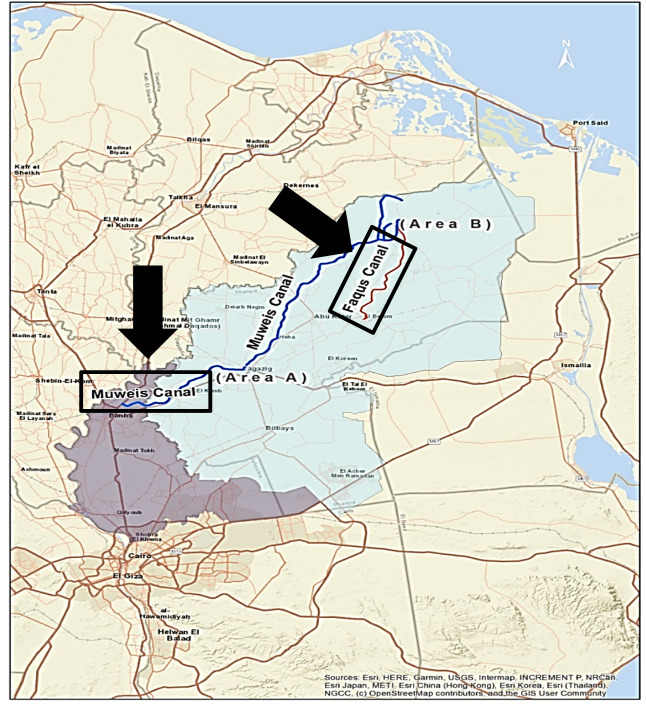
Location map of the study area

Al Sharkia governorate is located on the eastern side of the Nile Delta in Egypt and spans an area of 4911 km^2^. It was located between latitudes 30°42′0′′ and 30°42′0′′N and longitudes 31°48′0′′ and 31°48′0′′E (Fig. 1). In the research region, the overall length of the freshwater canal network is approximately 2729 km^2^. They were separated into main canals (Bahr Mowais and Ismailia Canals) and subordinate canals (Bahr Abo-Alakhdr and Bahr Faqous), which were further divided into smaller branches and canals. The surface water in the study area arises from AlRaiyah Al-Tawfky’s main canal that feeds the Bahr-Mowais Canal at 35 km^2^ and the Ismailia Canal from Al-Monier barrage at 28.15 km^2^ to El-Salhia lock at km^2^ of 75 (Ramadan et al. 2019). The fishing industries rely heavily on the selection of that location (El-Sayed et al. 2011; Samy-Kamal 2015).

### Sample’s collection

One hundred and twenty water and *Clarias gariepinus* fish samples weighing on average 279.52 ± 0.01 g and measuring on average 36.51 ± 1.7 cm in length were collected from two Al Sharkia government localities. Area A represents the Muweis canal in Zagazig city, which receives waste from the Miser oil and soap factory, and the San El-Hagar canal. Area B in Faqus city receives domestic and agricultural waste. The collected fish from the two areas were transported alive in their original water to the laboratory. *C. gariepinus* is the most consumed species in that studied area and is used as a bioindicator for those areas (Aly 2016). Samples were taken monthly over the four seasons from September 2017 to August 2018.

Water sample bottles were cleaned before being used with dilute nitric acid to remove any interfacial metal labels, then rinsed twice with distilled water before being used at each site. Monthly collected water samples were stored in an ice-containing isolated cooler and delivered to the laboratory the same day, where they were kept at 4 °C before processing and analysis.

After transporting the fish to the laboratory, they were measured for weight and length to determine body condition. The fish were dissected. The liver and gonads were removed and divided into three pieces, two of which were utilized to calculate heavy metal residues, oxidative stress, and antioxidant indicators. The third section was dedicated to histological analysis. If any parasites were found, they were fixed, kept, and identified in the manner described by (Madanire-Moyo and Barson 2010). The prevalence (average number of infected fish per examined fish), the intensity of infection (average number of parasites per infected fish), and the abundance (average number of parasites per examined fish) were calculated according to Rózsa et al. (2000).

### Heavy metals evaluation in water and fish (liver and gonads)

An atomic absorption spectrophotometer (Perkin Elmer, 2280) was used to detect the amounts of iron (Fe), copper (Cu), zinc (Zn), lead (Pb), manganese (Mn), and nickel (Ni) in water and fish samples. The samples were prepared and examined following the method of El-Shenawy et al. (2021b).

### Oxidative stress/antioxidant of catfish liver and gonads evaluation

Liver and gonad tissue homogenates were prepared and followed the kit’s instructions for determining the different oxidative stress/antioxidant parameters. According to the directions in the kit, lipid peroxidation (LPO) was measured using a colorimetric approach (Modesto and Martinez 2010). The thiobarbituric acid reactive product is formed when thiobarbituric acid combines with malondialdehyde in an acidic medium for 30 minutes at 95 °C. At 534 nm, the absorbance of the resulting pink product may be measured by a spectrophotometer.

The activity of superoxide dismutase (SOD) was measured spectrophotometrically at 560 nm, following the kit’s instructions and the Zikić et al. (2001) technique. The approach relied on the SOD enzyme’s capacity to prevent the nitroblue tetrazolium dye from being reduced by phenazine methosulphate.

The activity of catalase (CAT) was measured according to the kit’s instructions and the technique published by Atli et al. (2006). The CAT interacts with a defined amount of H_2_O_2_ and is halted by a CAT inhibitor after 1 min. In the presence of peroxidase, the remaining H_2_O_2_ reacts with 3,5-dichloro-2-hydroxybenzene sulfonic acid and 4-aminophenazone to form a chromophore with a color intensity inversely proportional to the amount of CAT in the sample. At 440 nm, the absorbance was measured.

Reduced glutathione (GSH) levels were measured according to the kit’s instructions, using an Atli and Canli (2008) approach based on the reductive cleavage of 5,5′-dithiobis (2-nitrobenzoic acid) by a sulfhydryl (-SH) group to produce a yellow hue. The GSH content is directly proportional to the decreased chromogen (absorbance measured at 412 nm).

### Parasite examination and identification according to Taha and Ramadan (2017)

Some Trematoda worms in the liver and gonads were fixed in 2.5% buffered glutaraldehyde (pH 7.4) made in 0.1 M sodium cacodylate at 4 °C. Post fixation treatment was carried out using 1% osmium tetroxide made in the same buffer at 4 °C for 1–2 h. Later, worms were dehydrated in ascending grades of ethanol, and then, the critical point was dried using carbon dioxide as a drying medium. Worms were then mounted on metallic stubs and coated with gold under vacuum conditions. The examination was carried out with a JEOL scanning electron microscope.

### Histological examination

For 24 h, an isolated worm, a portion of the catfish liver, and the gonads were fixed in 10% neutral buffered formalin. They were then regularly infiltrated in paraffin wax in a tissue processor and sectioned at 5 µm thickness. Mayer’s Hematoxylin and Eosin were used to stain it (Feldman and Wolfe 2014).

### Calculation and statistical analysis

#### Calculation

##### Fish’s general body condition

For the determination of the fish’s body condition (K), the weighed and length of the fish were measured. Fulton’s condition factor was performed according to Nash et al. (2006) as [weight/length^3^ × 100].

##### Transfer ratio (TR)

This ratio was used to compute the heavy metal transfer ratio: metal concentration in fish organ/metal concentration in water followed the formula of Zhang et al. (2019).

#### Statistical analysis

The analysis was carried out using SPSS version 20 (Statistical Package for the Social Sciences). The acquired findings were expressed as mean ± standard error. ANOVA was used, followed by a Duncan-significant difference test for the comparison between different treatments. ANOVA tests were considered statistically significantly different when *p* ≤ 0.05. Pearson rank correlation was applied to determine if there was a relationship between heavy metal accumulation in fish organs and parasite abundance.

## Results

### Fish’s general body condition

**Fig. 2:**
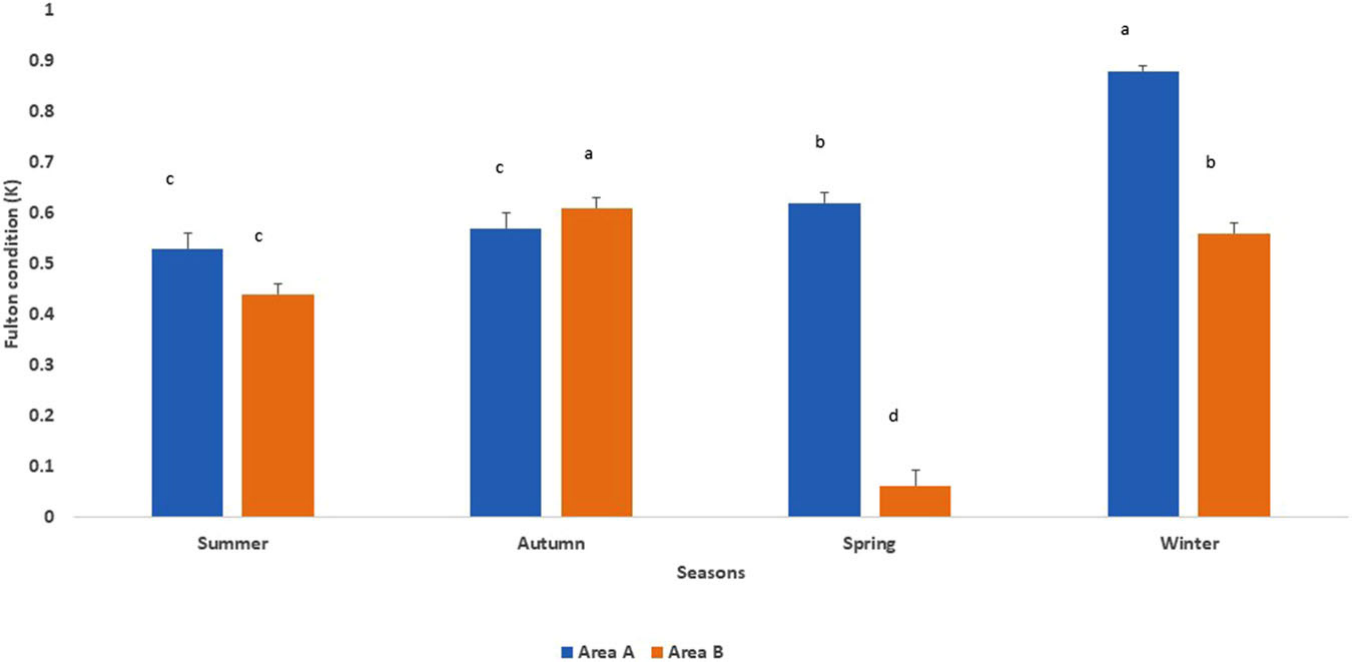
Seasonal variation in Fulton’s condition factors (K) of catfish species collected in two different areas of the Al Sharkia Government. The different letter is significant when *P* ≤ 0.05

The body condition of African catfish from the two areas of the Al Sharkia government was evaluated through Fulton’s condition factors (K) that were compared by season (Fig. 2). Seasonally, there were also significant (*P* ≤ 0.05) fluctuations in the average of K. In comparison to the other seasons, the mean condition factor was greater in the winter, with K = 0.88 ± 0.01 in region A, and the autumn, with 0.56 ± 0.02 in area B.

### Heavy metal determination in water samples

The concentration of seven heavy metals (Fe, Zn, Mn, Cu, Pb, Ni, and Cd) in water samples was calculated from the two regions during the investigation period as recorded in Table 1.

**Table 1:** Seasonal concentration of heavy metal (µg/L) in the water sample in the two areas

Area	Season	Fe	Zn	Mn	Cu	Pb	Ni	Cd
Area A	Summer	0.50 ± 0.01^c^	0.01 ± 0.01^c^	0.30 ± 0.01^d^	0.03 ± 0.01^a^	0.04 ± 0.01^b^	-	-
	Autumn	0.10 ± 0.01^a^	0.03 ± 0.01^a^	0.02 ± 0.01^ab^	-	-	-	-
	Winter	0.20 ± 0.01^a^	0.02 ± 0.01^a^	0.01 ± 0.01^a^	-	-	-	-
	Spring	2.30 ± 0.06^e^	0.05 ± 0.01^e^	0.03 ± 0.01^c^	0.01 ± 0.01^c^	0.03 ± 0.004^b^	0.14 ± 0.0	0.01 ± 0.001
Area B	Summer	0.60 ± 0.01^d^	0.02 ± 0.01^d^	0.17 ± 0.01^e^	0.04 ± 0.00^b^	0.03 ± 0.002^b^	-	-
	Autumn	0.40 ± 0.02^b^	0.01 ± 0.01^b^	0.01 ± 0.01^b^	-	0.02 ± 0.01^a^	-	-
	Winter	0.28 ± 0.03^b^	0.01 ± 0.003^b^	0.36 ± 0.004^c^	-	-	-	-
	Spring	-	-	-	-	-	-	-

The average of monthly samples (n = 15 for each season). Data presented as mean ± S.E. Different letter superscript is significant *P* ≤ 0.05. - is the data below the detection limit

In comparing the heavy metal in Area (A) during the four seasons. Area (A) showed a high concentration of iron (Fe), zinc (Zn), nickel (Ni), and cadmium (Cd) in spring and a high concentration of manganese (Mn), copper (Cu), and lead (Pb) in summer. Cd, Pb, Ni, and Cd disappeared completely in autumn and winter (Table 1). On the other hand, area (B) showed high concentrations of Fe and Mn during summer, autumn, and winter. Ni and Cd disappeared completely from all seasons. Iron (Fe) had the highest concentration in both areas. Fe concentration was in the following order: spring > summer > winter > autumn in area (A), while its concentration in area (B) was in the following order: summer > autumn > winter (Table 1).

One way analysis of heavy metals in water was done was to determine the differences between the four seasons in each area. The analysis indicated that there were significant differences between seasons for Fe, Zn, Mn, Cu, Pb, Ni, and Cd in two areas (Duncan, *P* ≤ 0.05) (Table 1).

### Heavy metal determination in fish (liver and gonads)

The concentration of six heavy metals (Fe, Zn, Mn, Cu, and Pb) in water samples was collected from the two regions during the investigation period as recorded in Table 2. One way analysis was done was to determine the differences between heavy metals in the liver and gonads during the four seasons in each area. The analysis indicated that there were significant differences between seasons for Fe, Zn, Mn, Cu, and Pb in the two areas (Duncan, *P* ≤ 0.05) (Table 2).

**Table 2:** Seasonal heavy metals level (µg/g) of the liver and gonads of *Clarias gariepinus* in the two studied areas

	Season	Organ	Fe	Zn	Mn	Cu	Pb
Area A	Summer	Liver	833.5 ± 44.3^d^	19.7 ± 1.0 ^f^	115.40 ± 10.15^b^	15.30 ± 1.50^b^	11.53 ± 0.88^b^
		Gonads	118.6 + ±2.1	111.5 ± 5.9	213.8 ± 22.0 ^c^	2.3 ± 0.3^d^	37.5 ± 1.0^c^
	Autumn	Liver	200.3 ± 5.4^a^	19.6 ± 1.5^a^	0.48 ± 0.05^a^	0.58 ± 0.10^a^	-
		Gonads	271.0 ± 13.3	13.3 ± 1.6	1.3 ± 0.2^a^	0.76 ± 0.01^b^	-
	Winter	Liver	342.0 ± 9.8^b,c^	8.7 ± 0.8^c,d^	1.11 ± 0.09^a^	6.3 ± 0.5^c,d^	-
		Gonads	178.5 ± 5.9	8.2 ± 0.6	3.2 ± 0.3^a^	5.0 ± 1.5^c^	-
	Spring	Liver	287.0 ± 42.6^c^	11.8 ± 0.1^e^	1.40 ± 0.06^a^	0.52 ± 0.01^a^	-
		Gonads	89.9 ± 10.7	23.9 ± 2.5	1.2 ± 0.1^a^	0.7 ± 0.1^b^	-
Area B	Summer	Liver	220.10 ± 9.5^a^	18.8 ± 2.1 ^g^	151.60 ± 2.89^c^	4.24 ± 0.7 ^g^	15.67 ± 2.85^c^
		Gonads	44.9 ± 5.2	24.8 ± 2.9	230.6 ± 8.3^b^	-	-
	Autumn	Liver	531.7 ± 43.3^a^	22.4 ± 1.0^d^	0.76 ± 0.07^a^	2.4 ± 0.36^d^	-
		Gonads	210.0 ± 12.8	46.1 ± 1.8	2.5 ± 0.4^a^	0.8 ± 0.02^b^	-
	Winter	Liver	598.4 ± 24.3^a,b^	18.7 ± 0.6^c^	0.87 ± 0.1^a^	10.3 ± 0.9^c^	-
		Gonads	158.8 ± 1.2	9.4 ± 0.1	17.1 ± 0.6^a^	5.3 ± 0.5^c^	-
	Spring	Liver	301.9 ± 24.5^a,b,c^	7.1 ± 0.3^b^	2.99 ± 0.4^a^	12.8 ± 0.6^b^	-
		Gonads	90.9 ± 6.9	4.2 ± 0.5	1.7 ± 0.3^a^	0.1 ± 0.01	0.2 ± 0.01^b^

The average of monthly samples. Data presented as mean ± S.E (n = 15 per season). Different letter superscript is significant at *P* ≤ 0.05. - is below the detection limit

In Area (A), Fe showed the highest concentration in the liver during the four seasons in the following order: summer > winter > spring > autumn. Lead (Pb) was found only in the liver during the summer in a low concentration. The concentration of the four heavy metals (Fe, Zn, Mn, and Cu) was in the following order: Fe > Zn > Cu > Mn during autumn and winter. Mn showed the highest concentration in the gonads of the infected fish in summer, while Fe showed the highest concentration during autumn, spring, and winter in the following order: autumn > winter > spring. Cu showed the lowest concentration in the gonads during the four seasons in the following order: winter > summer > autumn > spring (Table 2).

In Area (B), Fe had the highest concentration in the liver during the four seasons in the following order: winter > autumn > spring > summer. Mn showed the lowest concentration in the liver during autumn, spring, and winter. During autumn, winter, and spring, heavy metals are arranged in the following order: Fe > Zn > Cu > Mn. On the other hand, the highest concentration of Mn is found in the gonads during summer, and Cu is found with the lowest concentration during the four seasons in the following order: winter > autumn > spring. Pb is found only in the gonads during spring with a low concentration (Table 2).

### The transfer ratio of heavy metals

The transfer ratio (TR) of the heavy metals within catfish liver and gonads was given in Table 3. The data indicated that the highest value of TR of Fe in the livers of area A was in summer and winter, and the lowest was in the gonads of area A in spring. In area B, the highest value was in liver tissue in winter and autumn, and the lowest was in the gonads during spring. The TR of Zn in catfish organs showed the highest value in gonads of area A during summer and the lowest in the liver during spring. However, the TR of Zn in catfish organs in area B recorded the highest value in gonads during autumn and the lowest value in gonads during spring. The TR of Mn in the liver and gonads of catfish showed the highest value in gonads during summer and the lowest was in gonads in spring in area A.

**Table 3:** Seasonal transfer ratio of heavy metals (µg/g) in the liver and gonads of *Clarias gariepinus* to the bioaccumulation concentration of heavy metals in the water in the two studied areas

Area	Season	Organs	Fe	Zn	Mn	Cu	Pb
Area A	Summer	Liver	1595.1 ± 94.4^g^	987.1 ± 50.9	5214.0 ± 321^h^	436.7 ± 71.4	1442.0 ± 50.5^g^
		Gonads	223.5 ± 5.9^a^	6258.0 ± 491.8^e^	9532.0 ± 680.7^e^	53.7 ± 5.5^c^	5049 ± 198.3^e^
	Autumn	Liver	878.6 ± 23.5^a^	3562.0 ± 270.6	84.9 ± 9.6	-	-
		Gonads	1233.0 ± 63.5^b^	2423.0 ± 322.4^f^	215.9 ± 40.8^d^	-	-
	Winter	Liver	1522.4 ± 65.0	1587.0 ± 127.1^g^	61.1 ± 4.9	-	-
		Gonads	755.5 ± 13.4^d^	1508.0 ± 183.9^b^	175.5 ± 19.3^a^	-	-
	Spring	Liver	242.8 ± 34.1^b^	254.6 ± 1.6e	38.3 ± 2.4	4.1 ± 0.3	-
		Gonads	83.9 ± 8.7^c^	512.8 ± 57.9^d^	34.4 ± 2.3^g^	5.1 ± 0.5	-
Area B	Summer	Liver	401.7 ± 17.9^c^	1035.0 ± 130.4^a^	6941.0 ± 459.7^h^	98.8 ± 18.0	2030.0 ± 354.8^e,h^
		Gonads	85.02 ± 8.7^c^	1334 ± 173.5^b^	10377.0 ± 704.9^e^	-	2296 ± 82.4^f^
	Autumn	Liver	2414.7 ± 202.6^d^	4143.0 ± 424.3^f^	136.0 ± 11.6	-	-
		Gonads	928.5 ± 53.5^d^	7865.0 ± 469.8^e^	416.8 ± 58.8^d^	-	-
	Winter	Liver	2761.0 ± 101.2^d^	3465.0 ± 416.0^f^	46.3 ± 5.2	-	-
		Gonads	679.7 ± 37.9^d^	1529.0 ± 86.1^b^	923.9 ± 21.9	-	-
	Spring	Liver	255.8 ± 20.4^e^	166.0 ± 7.8	86.8 ± 13.6	84.2 ± 6.8	-
		Gonads	88.2 ± 5.6^c^	74.7 ± 10.8^c^	62.6 ± 7.6^c^	0.57 ± 0.04	9.7 ± 1.6

Data presented as mean ± S.E. Different letter superscript is significant *P* ≤ 0.05. (−) represent is the data below the detection limit

However, the TR of Mn in the liver and gonads of catfish of area B recorded the highest value in the liver during summer and the lowest value in was liver during winter. The TR of Cu in the liver of catfish in area A in summer showed the highest value and was not detected in the liver and gonad during the winter and autumn seasons.

However. in area B, the highest value was in the liver during summer duration and not detected in the liver and gonads during autumn and winter. The highest value of TR of Pb in area A is present in gonads during summer duration and not detected in the liver and gonads during spring, autumn, and winter. In area B, the highest value was in the liver and gonads during the summer season and was not detected in autumn and winter.

### Oxidative stress/antioxidant of catfish liver and gonads evaluation

Oxidative marker and antioxidant enzymes of liver and gonads in area A showed marked significant (*P* ≤ 0.05) difference in LPO, CAT, GSH, and SOD than that of area B (Table 4). On the other hand, there was a significant difference in MDA, CAT, GSH, and SOD activities in the area between the four seasons.

**Table 4:** Seasonal oxidative stress biomarker (µM/mg protein) in the liver and gonads of *Clarias gariepinus* in the two regions

Area	Season	Organs	Lipid peroxidation (µM/mg protein)	Catalase (µM/mg protein)	Reduced Glutathione (µM/mg protein)	Superoxide dismutase (µM/mg protein)
Area A	Summer	Liver	37.8 ± 0.74^b^	2.47 ± 0.03^a^	2.55 ± 0.2^e^	34.6 ± 1.2^h^
		Gonads	34.5 ± 2.82^c^	0.29 ± 0.01^e^	6.53 ± 0.8^b^	89.7 ± 3.9^g^
	Autumn	Liver	23.0 ± 1.3^d^	1.9 ± 0.2^b^	1.1 ± 0.0^f^	241.0 ± 6.2^d^
		Gonads	8.5 ± 0.35^g^	0.1 ± 0.01^f^	0.72 ± 0.1^g^	194.2 ± 25.1^e^
	Winter	Liver	11.0 ± 0^f^	0.9 ± 0^d^	1.2 ± 0.1^e^	280.0 ± 0.7^c^
		Gonads	14.0 ± 0.1^e^	1.8 ± 0^c^	15.0 ± 0.2^a^	1620.5 ± 79.9^a^
	Spring	Liver	19.88 ± 1.32^c^	1.85 ± 0.17^c^	4.98 ± 0.5^c^	168.6 ± 11.5^f^
		Gonads	43.0 ± 0.6^a^	0.1 ± 0^f^	2.7 ± 0.1^d^	463.0 ± 16.0^b^
Area B	Summer	Liver	82.8 ± 3.1^a^	2.36 ± 0.03^b^	6.29 ± 0.4^b^	110 ± 3.4
		Gonads	6.01 ± 0.7^f^	0.68 ± 0.0^c^	0.81 ± 0.0^h^	669.0 ± 2.7^d^
	Autumn	Liver	12.0 ± 0.2^d^	0.7 ± 0.0^b^	1.1 ± 0.1^f^	725 ± 6.1^c^
		Gonads	24.0 ± 1.4^b^	0.7 ± 0.0^b^	19.0 ± 1.1^a^	2244.62 ± 69.0^a^
	Winter	Liver	16.0 ± 1.8^c^	1.0 ± 0.1^c^	1.6 ± 0.2^e^	429 ± 4.0^e^
		Gonads	12.0 ± 1.6^d^	2.6 ± 0.4^a^	4.7 ± 0.0^d^	2148.0 ± 1.5^b^
	Spring	Liver	12.5 ± 2.1^c^	0.65 ± 0.02^d^	1.0 ± 0.02^g^	49.8 ± 3.2^g^
		Gonads	11.24 ± 0.2^e^	0.68 ± 0.0^c^	5.24 ± 0.68^c^	64.89 ± 12.5^f^

Data presented as mean ± S.E. of the average of monthly samples. Different letter superscript is significant *P* ≤ 0.05

### Parasite examination and identification

The current study found that 38 of the 121 examined fish were infected with *Acanthostomum absconditum* juvenile in the two studied areas of Al Sharkia Governorate, for a total infection of 31.40%, with a mean intensity of 7.63 and mean abundance of 13.81 (Table 5).

**Table 5:** Seasonal prevalence, intensity, and abundance of the parasite (*Acanthostomum absconditum*) in *Clarias gariepinus* at Al Sharkia Governorate

Area	Season	No. of examined fish	No. of infected fish	Total No. of parasites recovered	Prevalence %	Abundance	Intensity
Area A	Summer	20	-	-	-	-	-
	Autumn	14	6	24	42.9	17	4
	Winter	14	9	72	64.3	5.1	8
	Spring	13	1	3	7.7	0.23	3
Area B	Summer	14	2	12	14.2	0.9	6
	Autumn	15	9	96	60	6.4	10.7
	Winter	11	4	13	36.4	1.18	3.3
	Spring	20	7	70	35	3.5	10
	Total	121	38	290	31.4	13.8	7.6

Prevalence (%) = number of fish infected/number of fish examined ×100

Intensity = number of parasites/number of fish infected

Abundance = number of parasites/number of fish examined

- not detected

Table 5 summarized the seasonal prevalence of the recovered parasite (*A. absconditum*) in *Clarias gariepinus*. It was concluded that the highest rate of parasite infection was observed during the winter season, with infection rates of 64.3% in the area (A), followed by autumn (42.8%), while spring was recorded at 7.7% and summer showed no infection in the examined fish. Autumn had the highest rate of parasite infection in the area (B), with infection rates of 60%, followed by winter (36.3%) and spring (35%), with summer having the lowest rate of infection (14.2%).

The highest intensity rate of the parasite was recorded in autumn at area B (10.7), followed by spring (10), summer (6), and winter season (3.25). The lowest rate of intensity of the parasite was found in area A during spring.

Significance Pearson coefficient correlation between the heavy metal concentration in the liver and gonads of the catfish in the two studied areas with the parasite abundance (Tables 6 and 7) were found.

**Table 6:** Pearson coefficient correlation between the heavy metal concentration in the liver of the catfish in the two studied areas with the parasite abundance

Area (A)		Area (B)	
Heavy metal of liver (µg/g)	Parasite abundance	Heavy metal of water (mg/L)	Parasite abundance
Fe	−0.775*	Fe	0.178
Zn	0.897*	Zn	−0.342
Mn	0.938*	Mn	−0.247
Cu	0.932*	Cu	−0.252

* Is a significant difference *P* ≤ 0.01

**Table 7:** Pearson coefficient correlation between the heavy metal concentration in the gonad of the catfish in the two studied areas with the parasite abundance

Area (A)		Area (B)	
Heavy metal of gonads (µg/g)	Parasite abundance	Heavy metal of gonads (mg/l)	Parasite abundance
Fe	−0.04	Fe	−0.615*
Zn	−0.032	Zn	0.145
Mn	−0.109	Mn	0.113
Cu	−0.006	Cu	0.818*

* Is a significant difference *P* ≤ 0.01

**Fig. 3:**
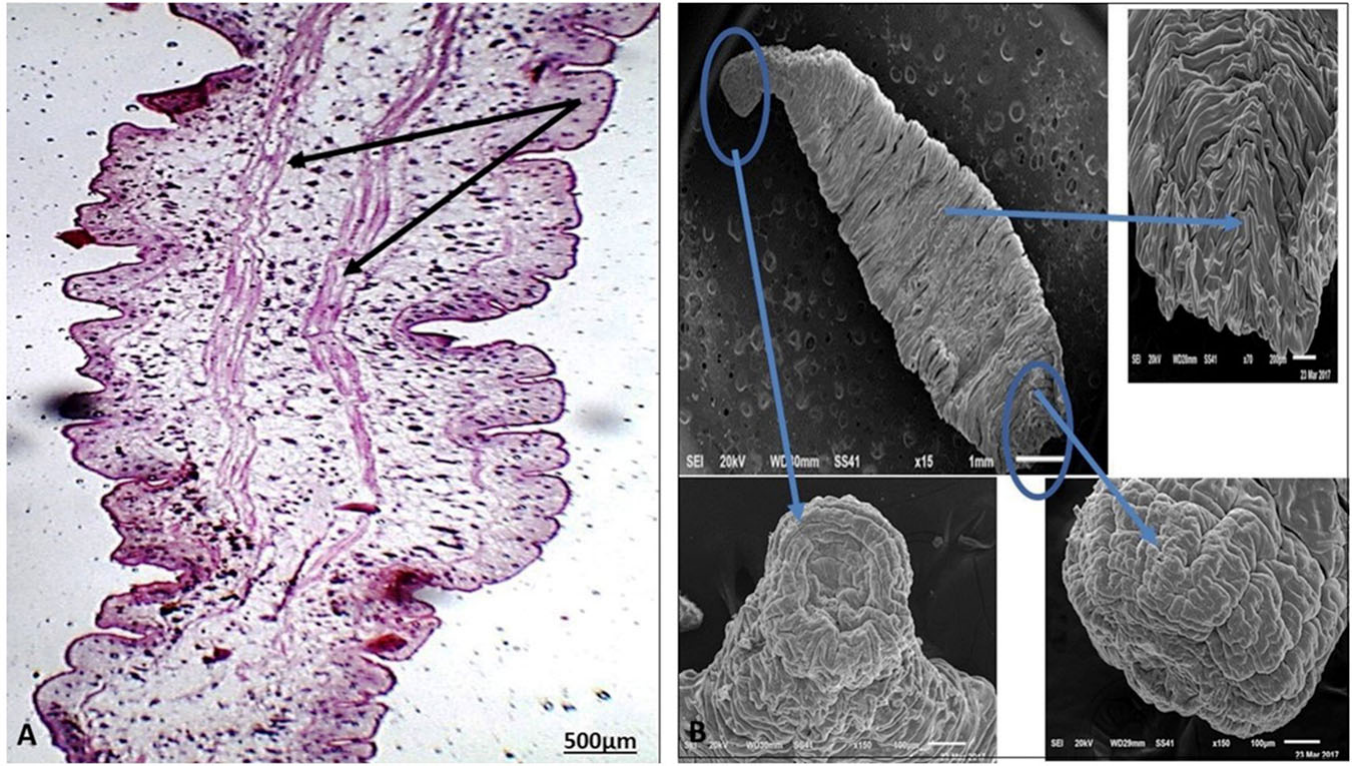
**A** Light micrographs showing whole mounts of juvenile *A. absconditum* stained with HE showed two intestinal caeca (arrow) in the middle portion of the body. **B** Scanning electron micrographs (SEM) of *A. absconditum* juveniles. a Ventral view of a juvenile stage-I showing that the circumoral tegmental crown is devoid of spines

Most of the parasites found are early juveniles of *A. absconditum* according to Ibraheem (2006) with their small body and a maximum length of about 0.8 mm. The oral sucker is terminal in position, circular in shape, and completely devoid of spines and the genital primordia are absent or not formed yet. The two intestinal caeca are observed and terminate posteriorly with two separate lateral anal openings (Fig. 3A). Scanning microscopy of the worm showed the oral sucker is terminal and rounded. Although the musculature of the oral sucker is well developed, the circumoral spines are absent (Fig. 3B).

### Histological examination

**Fig. 4:**
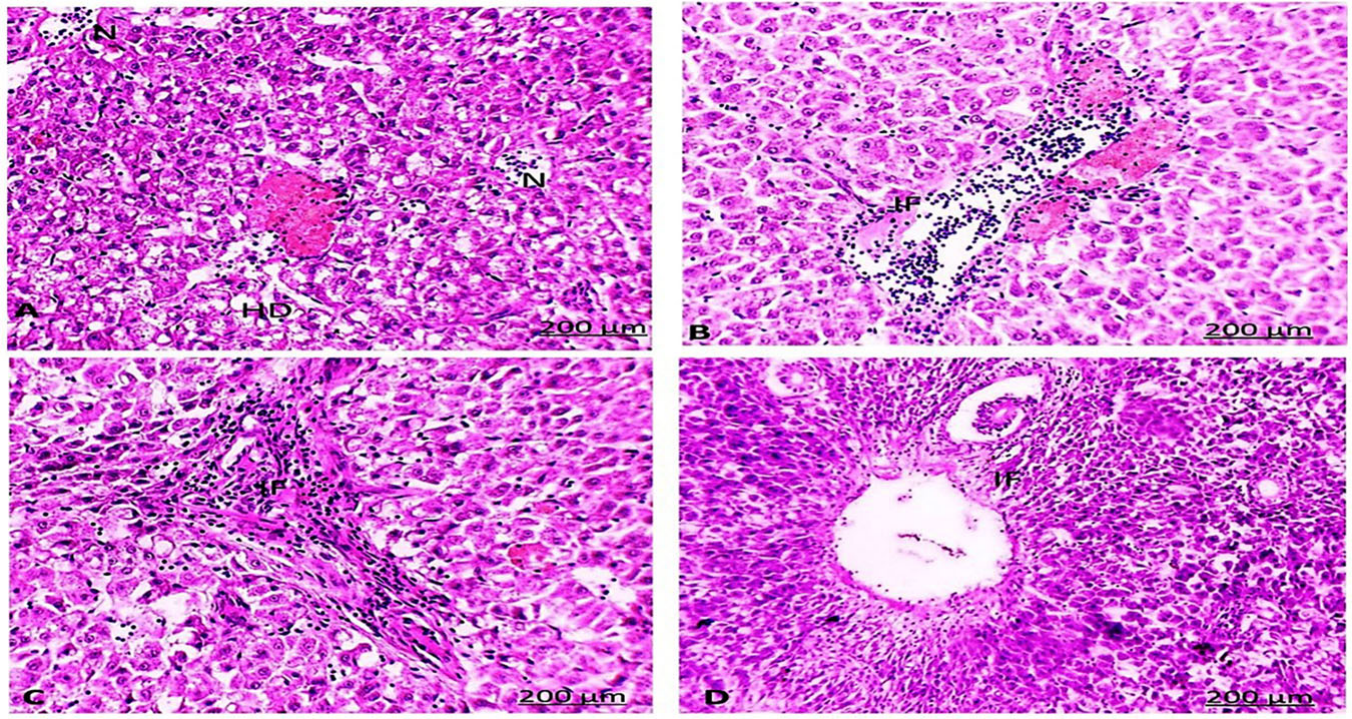
The liver of catfish in area A during summer (**A**) showed aggregations of inflammatory cells (IF) between the hepatocytes, thrombosis formation in a central vein, and hydropic degeneration (HD). Autumn (**B**) and spring (**C**) seasons showed aggregations of inflammatory cells (IF) between the hepatocytes. Winter (**D**) season showed an increase in IF between the hepatocytes (HE, 200X)

**Fig. 5:**
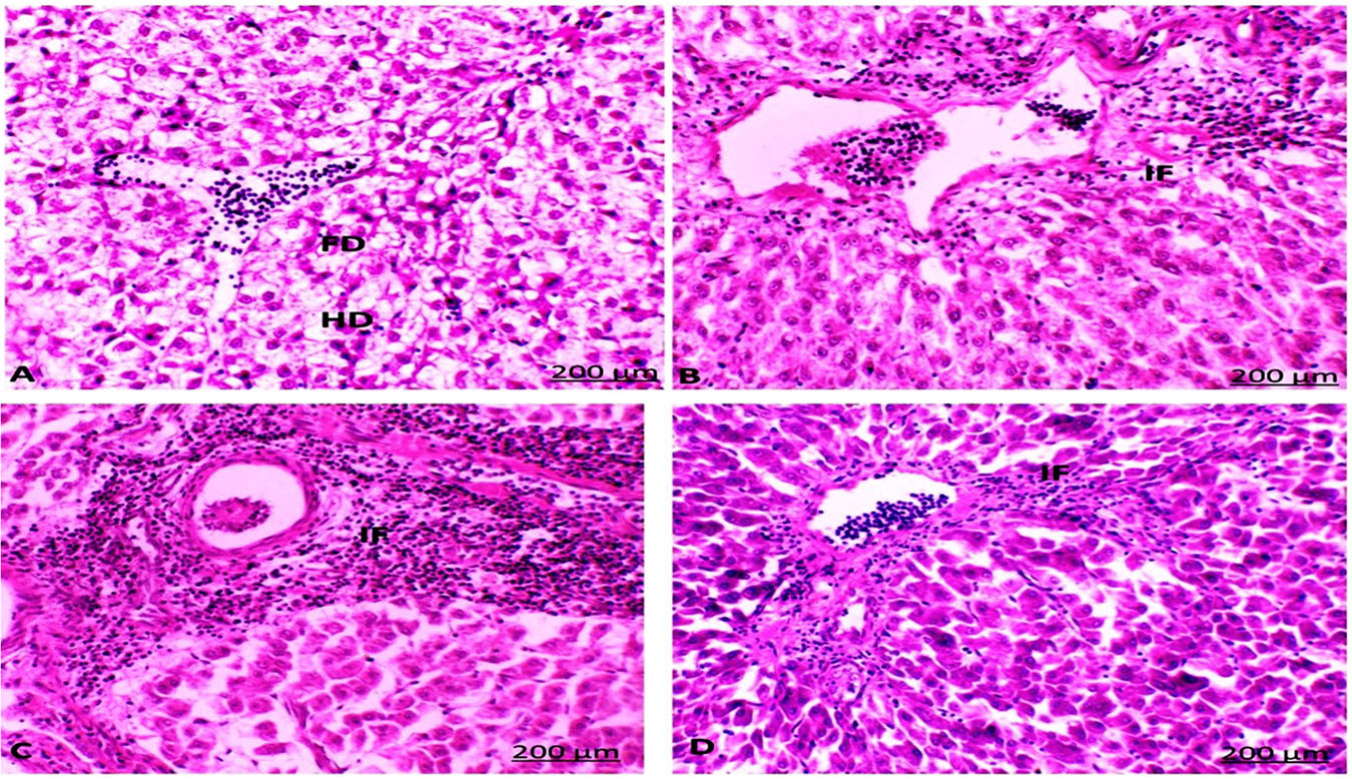
The liver of catfish in area B during the summer (**A**) season showed fatty (FD) and hydrobic (HD) degeneration. During autumn (**B**), spring (**C**) and winter (**D**) seasons showed aggregations of inflammatory cells (IF) between the hepatocytes (HE, 200X)

Seventy-eight percent of fish collected from area A and eighty percent of fish collected from area B during the four seasons were identified with different histological alterations detected in the liver and gonads (testes and ovaries) of *C. gariepinus* (Figs. 4 and 5, respectively).

**Fig. 6:**
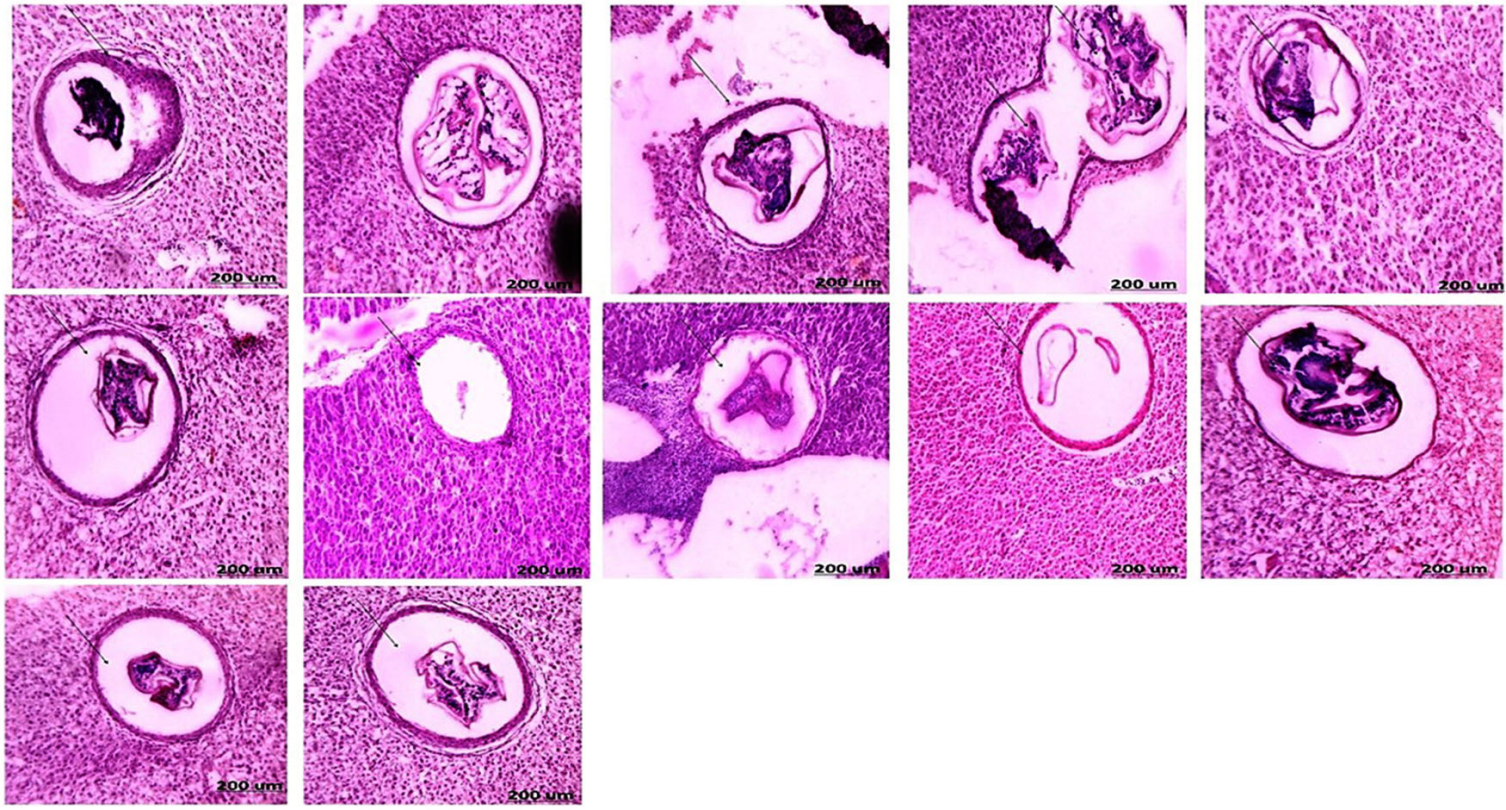
Liver section of catfish in area B during the four-season showed encysted metacercarial cysts (arrow) with irregular double layer wall and folded body (HE, 200X)

Congestion in the central and portal veins was a predominant feature in fish livers from the two sampling sites and was identified in catfish from areas A and B during the four seasons (Figs. 4 and 5). Numerous histopathological changes were found to be extensive in catfish during all seasons. Infiltrations of inflammatory cells were the most prevalent in livers compared to any of the other types of alterations identified especially with a response to encysted metacercaria (Fig. 6). With an irregular wall, the encysted metacercaria emerged. The cyst wall appeared round with two layers. Inside the cyst wall, there were clear refractive grains. The metacercaria’s body was folded, and the oral sucker was visible. Encysted metacercaria observed an increase in their number in the liver section of fish collected in the two areas during the winter season compared to the other seasons. Single necrotic and focal aggregated cell with pyknotic nucleus was identified in a high percentage of about 95% of the fish collected from area A during the four seasons (Fig. 4). Hydropic and fatty degeneration were identified in the liver of fish from area B during the four seasons (Fig. 4).

**Fig. 7:**
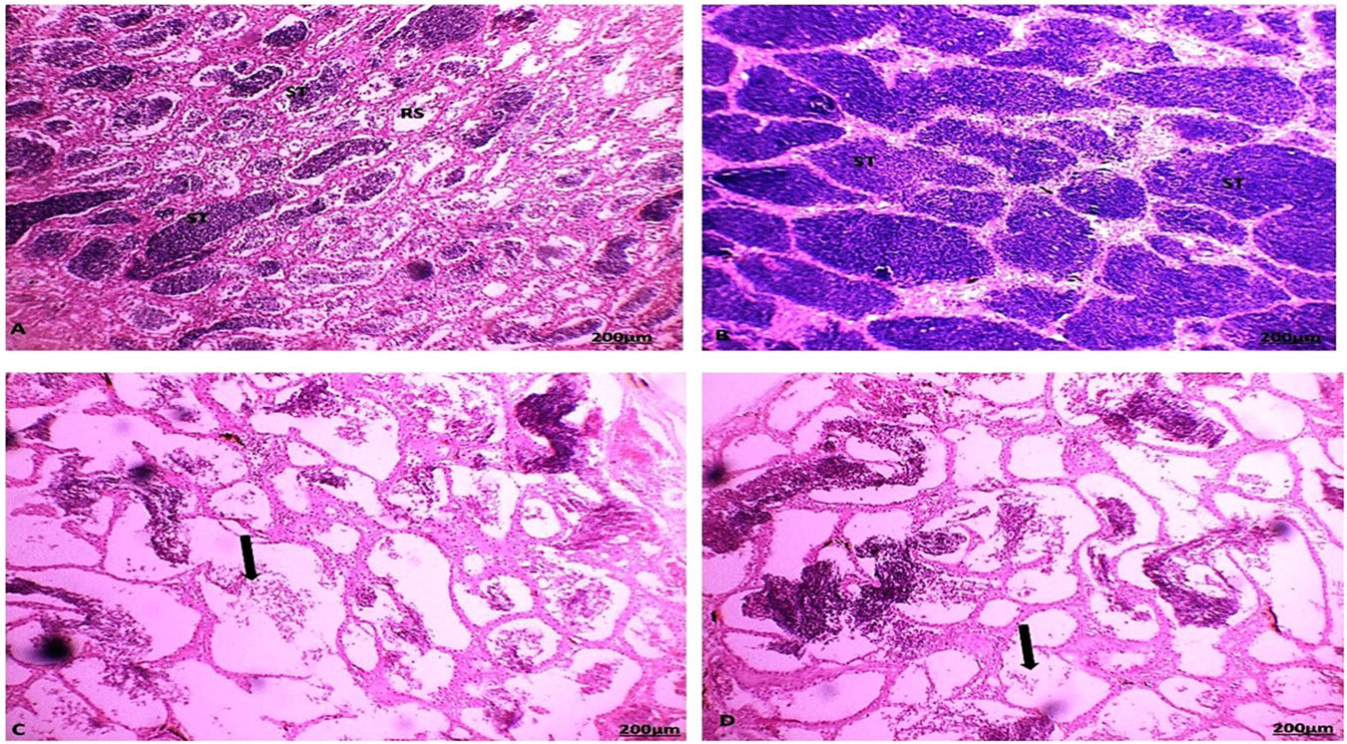
Section of catfish’s testis collected from area A during summer (**A**) showed testicular lobules (ST) with thin tunica albuginea and were filled with spermatozoa and some tubules showed residual spermatozoa (RS). Autumn (**B**) showed some testicular lobules (ST) with dense spermatozoa in the lumen. spring (**C**) showed testicular lobules decreased with all spermatogenic cells (arrow) and winter (**D**) showed some empty degenerated testicular lobules with all the developmental spermatogenic stages and thick tunica albuginea (arrow) (HE, 100X)

**Fig. 8:**
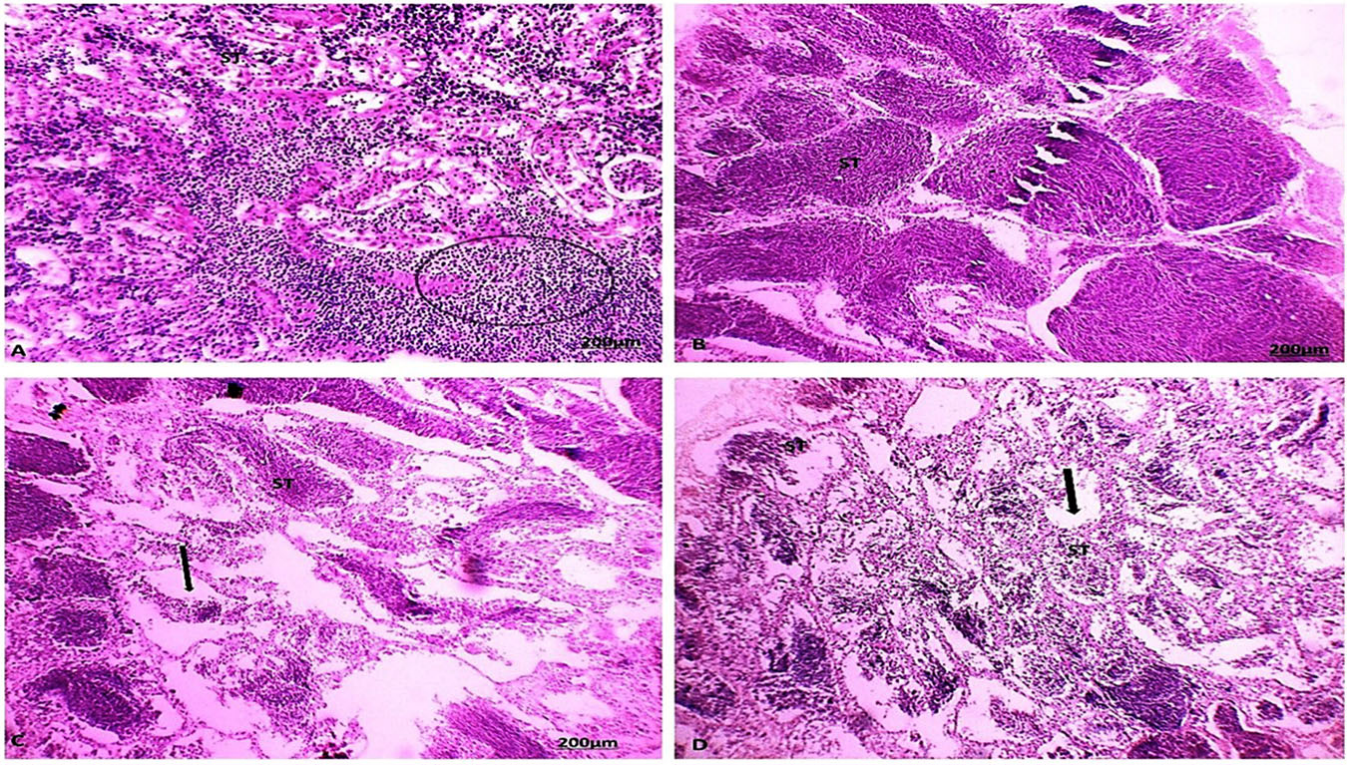
Section of catfish’s testis collected from area B during summer season (**A**) showed the absence of testicular lobules (ST) arrangement with infiltration of inflammatory cells between tubules (circle). Autumn season (**B**) showed testicular lobules (ST) with dense and increase spermatozoa in the lumen. Spring season (**C**) showed testicular lobules filled with all spermatogenic cells and some tubules with remnant spermatozoa (arrow) and the winter season (**D**) showed testicular lobules filled with all spermatogenic cells and some tubules with remnant spermatozoa (arrow) (HE, 100X)

During the summer season, the testes of catfish collected from area A showed seminiferous tubules with different spermatogenic stages and spermatozoa in the lumen of some lobules. As a result, the activity of the testicular seminiferous tubules varied, with most tubules distended with spermatozoa and some lobules appearing empty (spent lobules) because of their discharged spermatozoa (Fig. 7A). The testes of catfish collected from area B showed alteration in the histological structure of the testes with infiltration of inflammatory cells between the testicular tubules in response to the presence of encysted metacercaria (Fig. 8A). During the autumn season, the testes of catfish collected from areas A and B showed that interstitial connective tissue was normal. Spermatozoa increased in their thickness in the lumen of the seminiferous tubules (Figs. 7B and 8B). During the spring season, the testes of catfish collected from areas A and B showed both tunica albuginea and interstitial connective tissue were very thin and reduced due to the pressure exerted on them by the distended testicular lobules with different spermatogenic stages, with the appearance of the spermatozoa in the lumen of some lobules. Testicular lobules displayed varying levels of activity, with most lobules distended with spermatozoa and other lobules seeming empty because of spermatozoa discharge (Figs. 7C and 8C).

During the winter season, the testes of catfish collected from areas A and B showed a great increase in the connective tissue of both the tunica albuginea and interstitium. Most of the spermatogenic cells have degenerated except the spermatogonia, which appeared intact and predominant. Some testicular lobules contained residues of undischarged spermatozoa, while other lobules appeared empty (Figs. 7D and 8D).

**Fig. 9:**
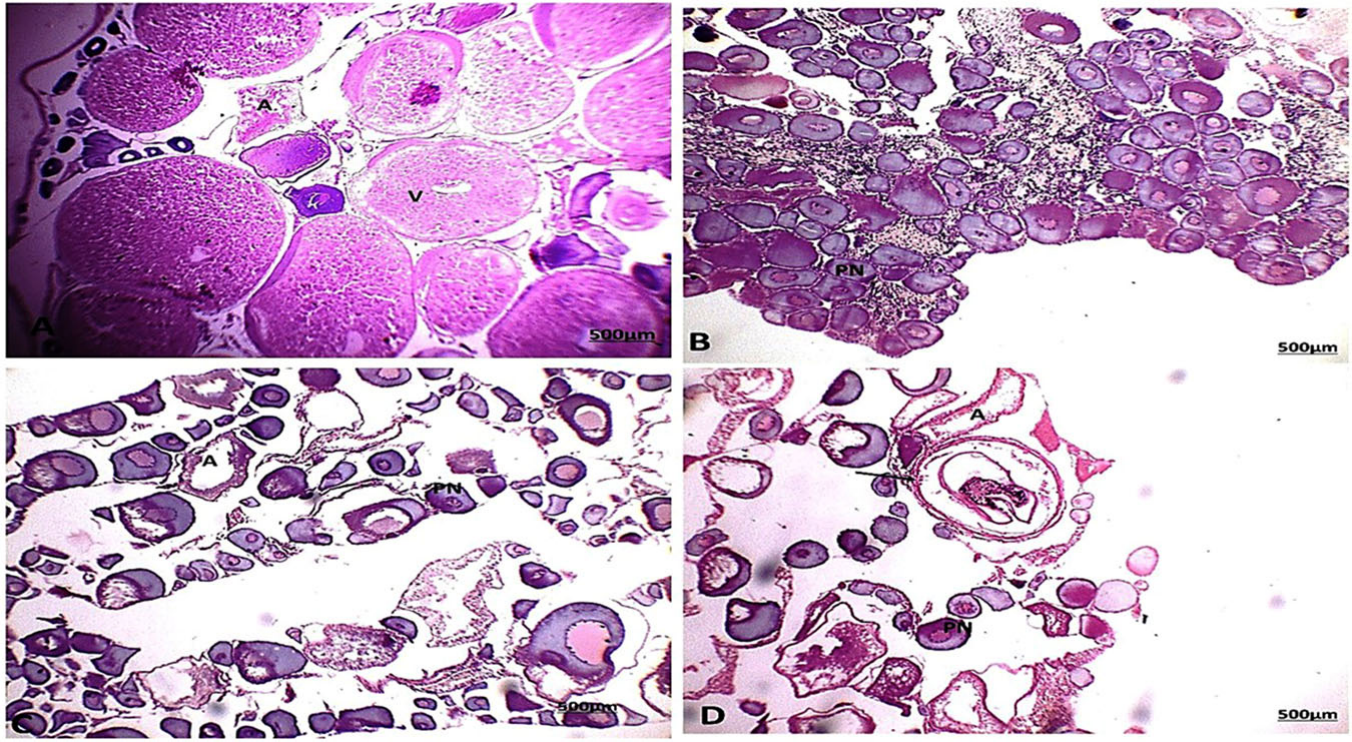
Section of catfish’s ovary in area A during summer season (**A**) showed abundant mature and vitellgenic follicles (V). during the autumn season (**B**) showed the predominance of the perinuclear stage (PN). during the spring season (**C**) showed a thickened wall of oogenic stages with the predominance of the perinuclear stage and atretic stage (A). during the winter season (**D**) showed perinuclear stage (PN), degenerated follicles, atretic stage (A), and encysted metacercaria (arrow) (HE, 100X)

**Fig. 10:**
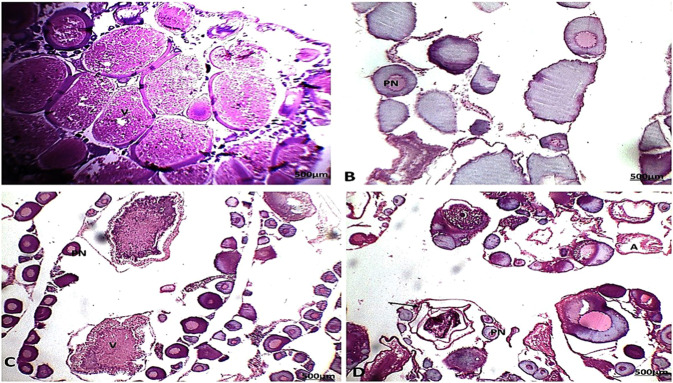
Section of catfish’s ovary in area B during summer season (**A**) showed abundant mature and vitellgenic follicles (V). during the autumn season (**B**) showed the predominance of the perinuclear stage (PN) and degenerated follicles. During spring season (**C**) showed a thickened wall of oogenic stages with the predominance of the perinuclear stage and vitellogenin stage (A). During the winter season (**D**) showed perinuclear stage (PN), degenerated follicles, atretic stage (A), and encysted metacercaria (arrow) (HE, 100X)

During the summer season, the ovary of catfish collected from areas A and B showed tunica albuginea becoming thinner (Figs. 9 and 10). The most predominant follicles were in vitellogenic stages. During the autumn season, the ovary of catfish collected from areas A and B showed that the tunica albuginea was relatively thicker. The most predominant stages were the perinucleus stage and previtellogenic follicles. During the spring season, the ovary of catfish collected from areas A and B of the tunica albuginea became thinner. The most predominant follicles were in the vitellogenic and perinucleus stages. The ovary of catfish collected from areas A and B showed the tunica albuginea surrounding the ovary reached a great thickness and the stromal connective tissue was increased. The ovarian structure revealed degenerated follicles and encysted metacercaria, but the previtellogenic stage was the most prevalent, with some follicles in the atretic stage.

## Discussion

This study examined the relationship between parasite communities and water quality in a variety of different aquatic environments. The environmental conditions in water bodies are constantly changed by various naturally and anthropogenically induced factors (Dodson and Hanazato 1995). Some organic pollutants accumulate preferentially in fatty tissues such as the liver and gonads of that fish, and the effects become apparent when levels in such tissues reach a threshold level (Authman et al. 2015). However, this accumulation depends upon their intake, storage, and elimination from the body (Ali et al. 2014). The current situation of the catfish is affected by the ecological disturbance in that aquatic environment (Barnhoorn et al. 2004).

Knowledge of Fulton’s condition factors (K) is very important as it is a good indicator of fish physiological condition and health. The present study also revealed seasonal variations in body condition in the African catfish from the two areas of the Al Sharkia government. In general, body condition was better in the winter. According to Lloret et al. (2002) Fulton’s variables, the fish’s physiological condition, and health may fluctuate in different environmental settings due to variances in water quality, feeding conditions, sex, stage of maturation, and the period during which the fish was taken. The K values of fish in the spring are significantly different in areas A and area B. These results may be due to the fluctuation of oxidative stress within the organ of the fish which negatively affected the fish’s health condition (Carminato et al. 2020).

Seasonally, the metal concentration in water samples from the two sites showed an uneven pattern throughout the year. Throughout the seasons, metal concentrations differed considerably (*P* ≤ 0.05) between the two regions. The quantity of agricultural drainage water, untreated home sewage, and industrial waste dumped into the canals and drains that feed the ponds might be the cause of these seasonal changes (Goher et al. 2014). Different authors have found seasonal fluctuations in metals in water at various water bodies in Al Sharkia (Al-Nagaaway et al. 2009; El-Sayed et al. 2011; Aly 2016; Mashaly et al. 2021).

African catfish prefer murky and foggy waters to dwell in. As a result of its relatively high-fat content, it is more susceptible to several sorts of environmental pollutants than other fish (Yahia and Elsharkawy 2014). African catfish can accumulate heavy metals in their tissue by absorption along the gill surface, kidney, liver, and gonads depending on metal type, the concentration of heavy metal in the water, and reproductive cycle (Authman et al. 2015). Seasonal variations in metal concentrations in the fish liver and gonads were considerable. These findings are consistent with those of Eastwood and Couture (2002) and Bahnasawy et al. (2011) who discovered seasonal fluctuations in metal content in fish organs and ascribed these variations to the amount of drainage water released into the drainage canal.

The bioavailability of metals may be influenced by the physiological activities of fish during different seasons (Qadir and Malik 2011). The findings demonstrated that throughout different seasons, the lowest concentration of all metals in both locations follows an erratic pattern. This agrees with Jakimska et al. (2011) who said that metal bioaccumulation in tissues varies depending on the metal. Furthermore, Van der Oost et al. (2003) proposed that pollutant accumulation patterns in fish and other aquatic animals are influenced by both absorption and clearance rates.

The liver plays an important role in detoxification and toxicant storage (Jaishankar et al. 2014). Due to its function, the liver contains a higher concentration of heavy metals than the gonads. The transfer ratio offered a way for determining heavy metal buildup in fish to determine the health risks to humans who ate the fish (Uysal et al. 2008). The identical accumulating tendency of the metals in fish, as well as their interactions, may explain the transfer ratio between them (Wang 2002).

The content of metals in water, the liver, and gonad tissues of fish samples revealed a strong link between them. The highest content of Fe in water was 2.3 µg/L in area A during spring. But the concentration of Fe in area A in spring in the liver was 287 µg/g, which was much lower than that in other seasons, a similar observation was observed in Zn and Cu. Fish in severely polluted areas acquire an enhanced state of oxidative stress, as evidenced by higher LPO levels and reduced antioxidant capacity in fish tissues (Padmini and Geetha 2009). Heavy metal accumulation altered the activity of antioxidant enzymes in the liver and gonads like SOD, CAT, and reduced GSH. Heavy metals may cause the inactivation and inhibitory effects of these enzymes. Overall, these effects may cause the cells to be exposed to oxidative attacks (Castro-González and Méndez-Armenta 2008). Farombi et al. (2007) showed reduced levels of antioxidant activity occurred in *C. gariepinus* in polluted areas contaminated with heavy metals.

Besides the contamination, 80% of fish disease results from parasitic infection (Sures 2006). The liver of the fish is used by the parasite as a haven to invade the fish’s immune system (Sitjà-Bobadilla 2008). The growth process of the parasite in the fish abdomen cavity caused a malfunction in the maturation of the gonads (parasitic sterility) that lead to a fish reduction (Parsa et al. 2011). Fish parasites can interact with contaminants in several ways (Vidal-Martinez et al. 2010). They can accumulate pollutants in their host organisms and serve as accumulation indicators (Palm 2011). This record may be attributed to the prolonged warm weather, which is the preferable condition for the parasitic cycle (Lafferty 2009). The current results reveal the presence of parasites in *C. gariepinus* from the two studied areas. Findings indicated a higher prevalence of catfish parasites in area B compared to area A. The greater incidence seen might be related to a variety of variables, including the eating habits of these fish, water pollution and heavy metals, and the availability of parasitic intermediate hosts (Afolabi et al. 2020). The present work showed a higher prevalence of catfish parasites and a low level of heavy metal accumulation in the winter and autumn season. The increase in the infection rate of fishes living in low levels of heavy metal accumulation to the fact that effluents including heavy metals could alter the availability or reduce the number of invertebrate intermediate hosts necessary for the life cycle of these parasites (Geeraerts and Belpaire 2010). The increased heavy metal concentrations may affect the abundance of the snail host resulting in fewer Trematoda parasites (Lefcort et al. 2002). The juveniles of *A. absconditum* were found to be more common in winter and autumn in areas A and B, respectively. Similar seasonal variations have been reported in other studies (Aly et al. 2020). Increasing transmission is probably due to reduced water volume, habitat contraction, and higher host and parasite densities (Wood et al. 2010).

The fish’s heavy metal accumulation, oxidative stress in their tissue, and suppression of their immune system made it at high risk of parasitic infection (Akinsanya et al. 2020). Changes in histopathological biomarkers in fish tissues such as the liver and gonads have received a lot of attention in assessing the effects of environmental stress and parasitic infection (Marigomez et al. 2006; Stentiford et al. 2003). Regarding the histopathological examination, the liver tissue of fish from areas A and B during the four seasons showed many alterations. Area B had marginally more alterations than area A. Both areas were found to be highly polluted. The exposure of fish to toxicants such as metals in the water in areas A and area B is likely to have caused histopathological alterations in the liver. Many publications have addressed the issue of histopathological alterations in the liver of African catfish after exposure to a pollutant (Karami et al. 2016).

The presence of pollutants and heavy metals is one of many environmental factors that can result in a dangerously affected catfish reproduction system (Authman et al. 2015). Mansour et al. (2018) reported that the environmental impacts of pollution caused a pronounced decline in the gonad activity of the studied fish, which was reflected clearly by decreasing sperm in ripe testes and ripe oocyte degeneration (atresia). In the present research, the results of histological examination of gonads proved that pollution disrupted the gonadal development, especially in the two-season (autumn and winter) and area B. It agrees with a study on fish living in polluted water conducted by Osman and Kloas (2010) and H Abdel-Kader and H Mourad (2019).

## Conclusion

The findings revealed that the most critical factor influencing the quality of the catfish in its native habitats is water pollution in the Ash Sharkia government. The present studies showed that the metal level of catfish liver and gonads fluctuates depending on the research location and capturing season. The parasitological and histological examinations indicate a significant presence in the liver and gonad tissue investigated. The results can be useful for the monitoring and health management of African catfish (*C. gariepinus*) populations. This might indicate that the water quality in the Al Sharkia government’s regions A and B is deteriorating compared to the standard water quality. Accordingly, great efforts must be exerted to reduce the number of pollutants in the water by subjecting the water to treatment that ensures its safety. While parasite infection did not reduce the pollutant load of their fish host, nor affected biotransformation processes, infection was associated with changes in the oxidative status.

## Data Availability

Data supporting findings are presented within the manuscript.

## References

[CR1] Adams SM, Giesy JP, Tremblay LA, Eason CT (2001). The use of biomarkers in ecological risk assessment: recommendations from the Christchurch Conference on Biomarkers in Ecotoxicology. Biomarkers.

[CR2] Afolabi OJ, Olususi FC, Odeyemi OO (2020). Comparative study of African catfish parasites from cultured and natural habitats. Bull Natl Res Centre.

[CR3] Akinsanya B, Ayanda IO, Fadipe AO, Onwuka B, Saliu JK (2020). Heavy metals, parasitologic and oxidative stress biomarker investigations in Heterotis niloticus from Lekki Lagoon, Lagos, Nigeria. Toxicol Rep.

[CR4] Al-Nagaaway A, Shehata M, Dawah A, Mansour E (2009). Evaluation of some heavy metals residues in different water sources and Nile tilapia in Sharkia Governorate Egypt. Egypt J Agric Res.

[CR5] Ali AS, US SA, Ahmad R (2014). Effect of different heavy metal pollution on fish. Res J Chem Env Sci.

[CR6] Aly MY (2016). Comparison of heavy metals levels in muscles, liver, and gills of three fish species collected from agricultural drainage water AT El-Abbassa fish farm, Sharkia, Egypt. Egypt J Aquat Biol Fish.

[CR7] Atli G, Alptekin Ö, Tükel S, Canli M (2006). Response of catalase activity to Ag^+^, Cd^2+^, Cr^6+^, Cu^2+^ and Zn^2+^ in five tissues of freshwater fish Oreochromis niloticus. Comp Biochem Physiol Part C: Toxicol Pharmacol.

[CR8] Atli G, Canli M (2008). Responses of metallothionein and reduced glutathione in a freshwater fish Oreochromis niloticus following metal exposures. Environ Toxicol Pharmacol.

[CR9] Authman MM, Zaki MS, Khallaf EA, Abbas HH (2015). Use of fish as bio-indicator of the effects of heavy metals pollution. J Aquac Res Dev.

[CR10] Bahnasawy M, Khidr AA, Dheina N (2011). Assessment of heavy metal concentrations in water, plankton, and fish of Lake Manzala, Egypt. Turk J Zool.

[CR11] Barnhoorn I, Bornman M, Pieterse G, Van Vuren J (2004). Histological evidence of intersex in feral sharptooth catfish (Clarias gariepinus) from an estrogen‐polluted water source in Gauteng, South Africa. Environ Toxicol Int J.

[CR12] Biller JD, Takahashi LS (2018). Oxidative stress and the fish immune system: phagocytosis and leukocyte respiratory burst activity. Anais Acad Bras Ciências.

[CR13] Carminato A, Pascoli F, Trocino A, Locatello L, Maccatrozzo L, Palazzi R, Radaelli G, Ballarin C, Bortoletti M, Bertotto D (2020). Productive results, oxidative stress and contaminant markers in European sea bass: conventional vs. organic feeding. Animals.

[CR14] Castro-González M, Méndez-Armenta M (2008). Heavy metals: Implications associated to fish consumption. Environ Toxicol Pharmacol.

[CR15] Dodson SI, Hanazato T (1995). Commentary on effects of anthropogenic and natural organic chemicals on development, swimming behavior, and reproduction of Daphnia, a key member of aquatic ecosystems. Environ Health Perspect.

[CR16] Eastwood S, Couture P (2002). Seasonal variations in condition and liver metal concentrations of yellow perch (Perca flavescens) from a metal-contaminated environment. Aquat Toxicol.

[CR17] El-Sayed E-S, Khater Z, El-Ayyat M, Nasr E-S (2011). Assessment of heavy metals in water, sediment and fish tissues, from, Sharkia province, Egypt. Egypt J Aquat Biol Fish.

[CR18] El-Shenawy NS, EL-Hak HNG, Ghobashy MA, Soliman MF, Mansour FA, Greish S (2021). Risk assessment of some heavy metals in two fish species *Oreochromis Niloticus* and *Clarias Gariepinus* from Sharqia province, Egypt. J Vet Med Res.

[CR19] El-Shenawy NS, Gad El-Hak HN, Ghobashy MA, Mansour FA, Soliman MFM (2021b) Using antioxidant changes in liver and gonads of Oreochromis niloticus as biomarkers for the assessment of heavy metals pollution at Sharkia province, Egypt Reg Stud Mar Sci:101863. 10.1016/j.rsma.2021.101863

[CR20] Elawady E, El Bayomi R, Darwish W, El-Atabany A (2019). Risk assessment of some heavy metals from Claris gariepinus (African catfish) consumed in Sharkia Governorate, Egypt. Zagazig. Vet J.

[CR21] Farombi EO, Adelowo O, Ajimoko Y (2007). Biomarkers of oxidative stress and heavy metal levels as indicators of environmental pollution in African catfish (Clarias gariepinus) from Nigeria Ogun River. Int J Environ Res Public Health.

[CR22] Farombi EO, Ajimoko YR, Adelowo OA (2008). Effect of butachlor on antioxidant enzyme status and lipid peroxidation in freshwater African catfish,(Clarias gariepinus). Int J Environ Res Public Health.

[CR23] Farrag D, Shehata S, Azab A (2019) Functional anatomy of the lips and buccopharyngeal cavity of siluroid fishes, Clarias gariepinus and Bagrus bajad Inhabiting Bahr Shebeen-Alkoom, Al-Menoufiya Governorate, Egypt. Egyptian Academic Journal of Biological Sciences, D. Histology & Histochemistry 9(1):21–34

[CR24] Feldman AT, Wolfe D (2014) Tissue processing and hematoxylin and eosin staining. In: Histopathology. Springer, p 31–43 10.1007/978-1-4939-1050-2_3 25015141

[CR25] Geeraerts C, Belpaire C (2010). The effects of contaminants in European eel: a review. Ecotoxicology.

[CR26] Goel P (2006) Water pollution: causes, effects, and control. New Age International

[CR27] Goher ME, Hassan AM, Abdel-Moniem IA, Fahmy AH, El-sayed SM (2014). Evaluation of surface water quality and heavy metal indices of Ismailia Canal, Nile River, Egypt. The Egyptian. J Aquat Res.

[CR28] Abdel-Kader HH, Mourad MH (2019). Bioaccumulation of heavy metals and physiological/histological changes in Gonads of Catfish (Clarias gariepinus) inhabiting Lake Maryout, Alexandria, Egypt. Egypt J Aquat Biol Fish.

[CR29] Hamid A, Aiyelaagbe O, Usman L, Ameen O, Lawal A (2010). Antioxidants: its medicinal and pharmacological applications. Afr J Pure Appl Chem.

[CR30] Hussien MT, El-Liethy MA, Abia ALK, Dakhil MA (2020). Low-cost technology for the purification of wastewater contaminated with pathogenic bacteria and heavy metals. Water, Air, Soil Pollut.

[CR31] Ibraheem MH (2006). On the morphology of Acanthostomum spiniceps (Looss, 1896) and A. absconditum (Looss, 1901)(Digenea: Cryptogonimidae: Acanthostominae) with particular reference to the juvenile stage. Acta Zool.

[CR32] Jaishankar M, Tseten T, Anbalagan N, Mathew BB, Beeregowda KN (2014). Toxicity, mechanism and health effects of some heavy metals. Interdiscip Toxicol.

[CR33] Jakimska A, Konieczka P, Skóra K, Namieśnik J (2011). Bioaccumulation of metals in tissues of marine animals, Part II: metal concentrations in animal tissues.. Pol J Environ Stud.

[CR34] Karami A, Romano N, Hamzah H, Simpson SL, Yap CK (2016). Acute phenanthrene toxicity to juvenile diploid and triploid African catfish (Clarias gariepinus): molecular, biochemical, and histopathological alterations. Environ Pollut.

[CR35] Lafferty KD (2009). The ecology of climate change and infectious diseases. Ecology.

[CR36] Lefcort H, Aguon M, Bond K, Chapman K, Chaquette R, Clark J, Kornachuk P, Lang B, Martin J (2002). Indirect effects of heavy metals on parasites may cause shifts in snail species compositions. Arch Environ Contam Toxicol.

[CR37] Lloret J, Gil de Sola L, Souplet A, Galzin R (2002). Effects of large-scale habitat variability on condition of demersal exploited fish in the north-western Mediterranean. ICES J Mar Sci.

[CR38] Luo P, Sun Y, Wang S, Wang S, Lyu J, Zhou M, Nakagami K, Takara K, Nover D (2020). Historical assessment and future sustainability challenges of Egyptian water resources management. J Clean Prod.

[CR39] M Aly S, El-Gheit A, Fadel A, Essam El-Din H (2020). Digentetic trematodes in dicentrarchus labrax cultured in egypt: prevalence, clinical features, body condition, and histopathology. Egypt J Aquat Biol Fish.

[CR40] Madanire-Moyo G, Barson M (2010). Diversity of metazoan parasites of the African catfish Clarias gariepinus (Burchell, 1822) as indicators of pollution in a subtropical African river system. J Helminthol.

[CR41] Mahboob S (2013). Environmental pollution of heavy metals as a cause of oxidative stress in fish: a review. Life Sci J.

[CR42] Mansour HAA, El-kady MAH, Almaaty AHA, Ramadan AM (2018). Effect of environmental pollution on gonads histology of the Nile Tilapia, Oreochromis niloticus from Lake Manzala, Egypt. Egypt J Aquat Biol Fish.

[CR43] Marigomez I, Soto M, Cancio I, Orbea A, Garmendia L, Cajaraville MP (2006). Cell and tissue biomarkers in mussel, and histopathology in hake and anchovy from Bay of Biscay after the Prestige oil spill (Monitoring Campaign 2003). Mar Pollut Bull.

[CR44] Mashaly MI, El-Naggar AM, El-Tantawy SA, Al-Gaafari SA (2021) Accumulation of nine heavy metals in water and gills, intestine and digenean parasites of the silver catfish, Bagrus bajad Forskål, 1775. J Parasit Dis 1–12 10.1007/s12639-020-01326-1 PMC8254677 34295048

[CR45] Modesto KA, Martinez CB (2010). Roundup® causes oxidative stress in liver and inhibits acetylcholinesterase in muscle and brain of the fish Prochilodus lineatus. Chemosphere.

[CR46] Nash RD, Valencia AH, Geffen AJ (2006). The origin of Fulton’s condition factor—setting the record straight. Fisheries.

[CR47] Osibona A, Kusemiju K, Akande G (2009). Fatty acid composition and amino acid profile of two freshwater species, African catfish (Clarias gariepinus) and tilapia (Tilapia zillii). Afr J Food, Agric, Nutr Dev.

[CR48] Osman AG, Kloas W (2010). Water quality and heavy metal monitoring in water, sediments, and tissues of the African Catfish Clarias gariepinus (Burchell, 1822) from the River Nile, Egypt. J Environ Prot.

[CR49] Padmini E, Geetha BV (2009). Impact of season on liver mitochondrial oxidative stress and the expression of HSP70 in grey mullets from contaminated estuary. Ecotoxicology.

[CR50] Palm HW (2011) Fish parasites as biological indicators in a changing world: can we monitor environmental impact and climate change? In: Progress in parasitology. Springer, p 223–250

[CR51] Pandey G, Madhuri S (2014). Heavy metals causing toxicity in animals and fishes. Res J Anim, Vet Fish Sci.

[CR52] Parsa KA, Mojazi AB, Sharifpour I, Jalali JB, Motalebi A (2011) Gonads tissue changes of Chalcalburnus mossulensis (Heckel, 1843) infected by Ligula intestinalis (cestoda). 85–94.

[CR53] Qadir A, Malik RN (2011). Heavy metals in eight edible fish species from two polluted tributaries (Aik and Palkhu) of the River Chenab, Pakistan. Biol Trace Element Res.

[CR54] Ramadan EM, Fahmy MR, Nosair AM, Badr AM (2019). Using geographic information system (GIS) modeling in evaluation of canals water quality in Sharkia Governorate, East Nile Delta, Egypt. Model Earth Syst Environ.

[CR55] Rózsa L, Reiczigel J, Majoros G (2000). Quantifying parasites in samples of hosts. J Parasitol.

[CR56] Samy-Kamal M (2015). Status of fisheries in Egypt: reflections on past trends and management challenges. Rev Fish Biol Fish.

[CR57] Sitjà-Bobadilla A (2008). Living off a fish: a trade-off between parasites and the immune system. Fish Shellfish Immunol.

[CR58] Soliman NF, Nasr SM, Okbah MA (2015). Potential ecological risk of heavy metals in sediments from the Mediterranean coast, Egypt. J Environ Health Sci Eng.

[CR59] Stentiford G, Longshaw M, Lyons B, Jones G, Green M, Feist S (2003). Histopathological biomarkers in estuarine fish species for the assessment of biological effects of contaminants. Mar Environ Res.

[CR60] Sures B (2003). Accumulation of heavy metals by intestinal helminths in fish: an overview and perspective. Parasitology.

[CR61] Sures B (2006). How parasitism and pollution affect the physiological homeostasis of aquatic hosts. J Helminthol.

[CR62] Sures B, Nachev M, Selbach C, Marcogliese DJ (2017). Parasite responses to pollution: what we know and where we go in ‘Environmental Parasitology’. Parasites Vectors.

[CR63] Taha RG, Ramadan MM (2017). Scanning Electron Microscope of Sclerodistomum Egyptian n. sp.(Digenea, Sclerodistomidae) from the Marine Fish Saurida undosquamis from the Suez Gulf, Red Sea, Egypt. Egypt J Aquat Biol Fish.

[CR64] Uysal K, Emre Y, Köse E (2008). The determination of heavy metal accumulation ratios in muscle, skin and gills of some migratory fish species by inductively coupled plasma-optical emission spectrometry (ICP-OES) in Beymelek Lagoon (Antalya/Turkey). Microchem J.

[CR65] Van der Oost R, Beyer J, Vermeulen NP (2003). Fish bioaccumulation and biomarkers in environmental risk assessment: a review. Environ Toxicol Pharmacol.

[CR66] Vidal-Martinez VM, Pech D, Sures B, Purucker ST, Poulin R (2010). Can parasites really reveal environmental impact?. Trends Parasitol.

[CR67] Wang W-X (2002). Interactions of trace metals and different marine food chains. Mar Ecol Prog Ser.

[CR68] Wood CL, Lafferty KD, Micheli F (2010). Fishing out marine parasites? Impacts of fishing on rates of parasitism in the ocean. Ecol Lett.

[CR69] Yahia D, Elsharkawy EE (2014). Multi pesticide and PCB residues in Nile tilapia and catfish in Assiut city, Egypt. Sci Total Environ.

[CR70] Yancheva V, Velcheva I, Stoyanova S, Georgieva E (2016). Histological biomarkers in fish as a tool in ecological risk assessment and monitoring programs: a review. Appl Ecol Environ Res.

[CR71] Zhang Y, Feng J, Gao Y, Liu X, Qu L, Zhu L (2019). Physiologically based toxicokinetic and toxicodynamic (PBTK-TD) modelling of Cd and Pb exposure in adult zebrafish Danio rerio: accumulation and toxicity. Environ Pollut.

[CR72] Zikić R, Stajn AS, Pavlović SZ, Ognjanović BI, Saićić Z (2001). Activities of superoxide dismutase and catalase in erythrocytes and plasma transaminases of goldfish (Carassius auratus gibelio Bloch.) exposed to cadmium. Physiol Res.

